# Supplementary material for: Modeling the Seasonal Variation of Windborne Transmission of Porcine Reproductive and Respiratory Syndrome Virus between Swine Farms
Source: Viruses. 2023 Aug 18;15(8):1765. doi: 10.3390/v15081765 (PMC10459243; doi:10.3390/v15081765)
Supplement: Supplementary file 1 [file viruses-15-01765-s001.zip › File S2 ASCDATA_file.pdf]

ASCDATA.CFG

The model implementation utilized the sample ASCDATA.CFG file. For a comprehensive understanding of the parameter's definition, please refer to the HYSPLIT user guide provided at the following link: https://www.ready.noaa.gov/hysplitusersguide/S444.htm

```
-90.0  -180.0
1.0  1.0
180  360
2
0.2
'.'
```
