# Supplementary material for: Modeling the Seasonal Variation of Windborne Transmission of Porcine Reproductive and Respiratory Syndrome Virus between Swine Farms
Source: Viruses. 2023 Aug 18;15(8):1765. doi: 10.3390/v15081765 (PMC10459243; doi:10.3390/v15081765)
Supplement: Supplementary file 1 [file viruses-15-01765-s001.zip › File S3 CONTROL_file.pdf]

CONTROL file

The model implementation utilized the sample Control file. For a comprehensive understanding of the parameter's definition, please refer to the HYSPLIT user guide provided at the following link:
https://www.ready.noaa.gov/hysplitusersguide/S310.htm

```
0 0 0 0
1
40.0 -90.0 50.0
48
0
10000
1
met_data
met_data_name
1
part
1.0
1.0
0 0 0 0
1
40.0 -90.0
1.0 1.0
180.0 260.0
./
output.bin
1
50
0 0 0 0
1 0 0 0
0 24 0
1
0 0 0
0 0 0 0 0
0 0 0
0
0
/
```
