# Supplementary material for: Modeling the Seasonal Variation of Windborne Transmission of Porcine Reproductive and Respiratory Syndrome Virus between Swine Farms
Source: Viruses. 2023 Aug 18;15(8):1765. doi: 10.3390/v15081765 (PMC10459243; doi:10.3390/v15081765)
Supplement: Supplementary file 1 [file viruses-15-01765-s001.zip › Table_S1.pdf]

Table S1.

The table represents a total of 259 outbreak instances, referring to cases from emitting farms, observed over a 5-year study period from 15th January 2014 to 26th December 2018.

Case	Week 1	Week 2	Number of Participant Farms	No. Infected and Excreting Farms	No. Non-Infected Susceptible Farm	Week 3	Newly Infected Excreting Farms on week 3
1	1-Jan-2014	8-Jan-2014	167	28	139	15-Jan-2014	0
2	8-Jan-2014	15-Jan-2014	167	28	139	22-Jan-2014	0
3	15-Jan-2014	22-Jan-2014	167	28	139	29-Jan-2014	0
4	22-Jan-2014	29-Jan-2014	167	28	139	5-Feb-2014	0
5	29-Jan-2014	5-Feb-2014	167	29	138	12-Feb-2014	0
6	5-Feb-2014	12-Feb-2014	167	29	138	19-Feb-2014	0
7	12-Feb-2014	19-Feb-2014	167	29	138	26-Feb-2014	0
8	19-Feb-2014	26-Feb-2014	167	29	138	5-Mar-2014	0
9	26-Feb-2014	5-Mar-2014	167	29	138	12-Mar-2014	0
10	5-Mar-2014	12-Mar-2014	167	29	138	19-Mar-2014	0
11	12-Mar-2014	19-Mar-2014	167	29	138	26-Mar-2014	0
12	19-Mar-2014	26-Mar-2014	167	29	138	2-Apr-2014	0
13	26-Mar-2014	2-Apr-2014	167	33	134	9-Apr-2014	0
14	2-Apr-2014	9-Apr-2014	167	32	135	16-Apr-2014	0
15	9-Apr-2014	16-Apr-2014	167	32	135	23-Apr-2014	0
16	16-Apr-2014	23-Apr-2014	167	31	136	30-Apr-2014	0
17	23-Apr-2014	30-Apr-2014	167	29	138	7-May-2014	0
18	30-Apr-2014	7-May-2014	167	28	139	14-May-2014	0
19	7-May-2014	14-May-2014	167	28	138	21-May-2014	1
20	14-May-2014	21-May-2014	167	28	139	28-May-2014	0
21	21-May-2014	28-May-2014	167	25	141	4-Jun-2014	1
22	28-May-2014	4-Jun-2014	167	26	141	11-Jun-2014	0
23	4-Jun-2014	11-Jun-2014	167	26	140	18-Jun-2014	1
24	11-Jun-2014	18-Jun-2014	167	27	139	25-Jun-2014	1
25	18-Jun-2014	25-Jun-2014	167	28	139	2-Jul-2014	0
26	25-Jun-2014	2-Jul-2014	167	26	140	9-Jul-2014	1
27	2-Jul-2014	9-Jul-2014	167	26	140	16-Jul-2014	1
28	9-Jul-2014	16-Jul-2014	167	26	141	23-Jul-2014	0
29	16-Jul-2014	23-Jul-2014	167	24	143	30-Jul-2014	0
30	23-Jul-2014	30-Jul-2014	167	23	143	6-Aug-2014	1
31	30-Jul-2014	6-Aug-2014	167	24	143	13-Aug-2014	0
32	6-Aug-2014	13-Aug-2014	167	23	144	20-Aug-2014	0
33	13-Aug-2014	20-Aug-2014	167	21	146	27-Aug-2014	0
34	20-Aug-2014	27-Aug-2014	167	20	147	3-Sep-2014	0
35	27-Aug-2014	3-Sep-2014	167	19	148	10-Sep-2014	0
36	3-Sep-2014	10-Sep-2014	167	19	148	17-Sep-2014	0
37	10-Sep-2014	17-Sep-2014	167	19	148	24-Sep-2014	0
38	17-Sep-2014	24-Sep-2014	167	19	148	1-Oct-2014	0
39	24-Sep-2014	1-Oct-2014	167	19	148	8-Oct-2014	0
40	1-Oct-2014	8-Oct-2014	167	18	148	15-Oct-2014	1
41	8-Oct-2014	15-Oct-2014	167	17	150	22-Oct-2014	0
42	15-Oct-2014	22-Oct-2014	167	16	151	29-Oct-2014	0
43	22-Oct-2014	29-Oct-2014	167	16	150	5-Nov-2014	1
44	29-Oct-2014	5-Nov-2014	167	17	150	12-Nov-2014	0
45	5-Nov-2014	12-Nov-2014	167	17	150	19-Nov-2014	0
46	12-Nov-2014	19-Nov-2014	167	17	149	26-Nov-2014	1
47	19-Nov-2014	26-Nov-2014	167	18	149	3-Dec-2014	0
48	26-Nov-2014	3-Dec-2014	167	18	147	10-Dec-2014	2
49	3-Dec-2014	10-Dec-2014	167	20	146	17-Dec-2014	1
50	10-Dec-2014	17-Dec-2014	167	21	145	24-Dec-2014	1

51	17-Dec-2014	24-Dec-2014	167	22	145	31-Dec-2014	0
52	24-Dec-2014	31-Dec-2014	167	22	145	7-Jan-2015	0
53	31-Dec-2014	7-Jan-2015	167	22	145	14-Jan-2015	0
54	7-Jan-2015	14-Jan-2015	167	22	145	21-Jan-2015	0
55	14-Jan-2015	21-Jan-2015	167	22	145	28-Jan-2015	0
56	21-Jan-2015	28-Jan-2015	167	21	146	4-Feb-2015	0
57	28-Jan-2015	4-Feb-2015	167	21	146	11-Feb-2015	0
58	4-Feb-2015	11-Feb-2015	167	21	145	18-Feb-2015	1
59	11-Feb-2015	18-Feb-2015	167	22	145	25-Feb-2015	0
60	18-Feb-2015	25-Feb-2015	167	21	146	4-Mar-2015	0
61	25-Feb-2015	4-Mar-2015	167	21	146	11-Mar-2015	0
62	4-Mar-2015	11-Mar-2015	167	20	146	18-Mar-2015	1
63	11-Mar-2015	18-Mar-2015	167	21	146	25-Mar-2015	0
64	18-Mar-2015	25-Mar-2015	167	21	144	1-Apr-2015	2
65	25-Mar-2015	1-Apr-2015	167	23	144	8-Apr-2015	0
66	1-Apr-2015	8-Apr-2015	167	23	140	15-Apr-2015	4
67	8-Apr-2015	15-Apr-2015	167	27	140	22-Apr-2015	0
68	15-Apr-2015	22-Apr-2015	167	27	139	29-Apr-2015	1
69	22-Apr-2015	29-Apr-2015	167	28	138	6-May-2015	1
70	29-Apr-2015	6-May-2015	167	27	140	13-May-2015	0
71	6-May-2015	13-May-2015	167	27	140	20-May-2015	0
72	13-May-2015	20-May-2015	167	27	139	27-May-2015	1
73	20-May-2015	27-May-2015	167	28	139	3-Jun-2015	0
74	27-May-2015	3-Jun-2015	167	28	138	10-Jun-2015	1
75	3-Jun-2015	10-Jun-2015	167	28	139	17-Jun-2015	0
76	10-Jun-2015	17-Jun-2015	167	28	139	24-Jun-2015	0
77	17-Jun-2015	24-Jun-2015	167	28	138	1-Jul-2015	1
78	24-Jun-2015	1-Jul-2015	167	28	133	8-Jul-2015	6
79	1-Jul-2015	8-Jul-2015	167	33	133	15-Jul-2015	1
80	8-Jul-2015	15-Jul-2015	167	31	135	22-Jul-2015	1
81	15-Jul-2015	22-Jul-2015	167	32	135	29-Jul-2015	0
82	22-Jul-2015	29-Jul-2015	167	30	137	5-Aug-2015	0
83	29-Jul-2015	5-Aug-2015	167	30	135	12-Aug-2015	2
84	5-Aug-2015	12-Aug-2015	167	32	135	19-Aug-2015	0
85	12-Aug-2015	19-Aug-2015	167	32	135	26-Aug-2015	0
86	19-Aug-2015	26-Aug-2015	167	32	135	2-Sep-2015	0
87	26-Aug-2015	2-Sep-2015	167	32	135	9-Sep-2015	0
88	2-Sep-2015	9-Sep-2015	167	32	134	16-Sep-2015	1
89	9-Sep-2015	16-Sep-2015	167	33	134	23-Sep-2015	0
90	16-Sep-2015	23-Sep-2015	167	33	134	30-Sep-2015	0
91	23-Sep-2015	30-Sep-2015	167	33	134	7-Oct-2015	0
92	30-Sep-2015	7-Oct-2015	167	32	135	14-Oct-2015	0
93	7-Oct-2015	14-Oct-2015	167	32	135	21-Oct-2015	0
94	14-Oct-2015	21-Oct-2015	167	27	139	28-Oct-2015	1
95	21-Oct-2015	28-Oct-2015	167	28	137	4-Nov-2015	2
96	28-Oct-2015	4-Nov-2015	167	30	136	11-Nov-2015	1
97	4-Nov-2015	11-Nov-2015	167	31	133	18-Nov-2015	3
98	11-Nov-2015	18-Nov-2015	167	34	132	25-Nov-2015	1
99	18-Nov-2015	25-Nov-2015	167	35	131	2-Dec-2015	1
100	25-Nov-2015	2-Dec-2015	167	36	128	9-Dec-2015	3
101	2-Dec-2015	9-Dec-2015	167	37	129	16-Dec-2015	1
102	9-Dec-2015	16-Dec-2015	167	38	128	23-Dec-2015	1
103	16-Dec-2015	23-Dec-2015	167	39	128	30-Dec-2015	0
104	23-Dec-2015	30-Dec-2015	167	39	126	6-Jan-2016	2
105	30-Dec-2015	6-Jan-2016	167	40	126	13-Jan-2016	1
106	6-Jan-2016	13-Jan-2016	167	41	125	20-Jan-2016	1
107	13-Jan-2016	20-Jan-2016	167	42	124	27-Jan-2016	1
108	20-Jan-2016	27-Jan-2016	167	43	124	3-Feb-2016	0
109	27-Jan-2016	3-Feb-2016	167	43	124	10-Feb-2016	0
110	3-Feb-2016	10-Feb-2016	167	43	124	17-Feb-2016	0

111	10-Feb-2016	17-Feb-2016	167	42	124	24-Feb-2016	1
112	17-Feb-2016	24-Feb-2016	167	42	125	2-Mar-2016	0
113	24-Feb-2016	2-Mar-2016	167	42	123	9-Mar-2016	2
114	2-Mar-2016	9-Mar-2016	167	44	121	16-Mar-2016	2
115	9-Mar-2016	16-Mar-2016	167	46	121	23-Mar-2016	0
116	16-Mar-2016	23-Mar-2016	167	46	121	30-Mar-2016	0
117	23-Mar-2016	30-Mar-2016	167	46	120	6-Apr-2016	1
118	30-Mar-2016	6-Apr-2016	167	46	120	13-Apr-2016	1
119	6-Apr-2016	13-Apr-2016	167	42	124	20-Apr-2016	1
120	13-Apr-2016	20-Apr-2016	167	43	124	27-Apr-2016	0
121	20-Apr-2016	27-Apr-2016	167	43	124	4-May-2016	0
122	27-Apr-2016	4-May-2016	167	42	125	11-May-2016	0
123	4-May-2016	11-May-2016	167	40	126	18-May-2016	1
124	11-May-2016	18-May-2016	167	39	128	25-May-2016	0
125	18-May-2016	25-May-2016	167	39	127	1-Jun-2016	1
126	25-May-2016	1-Jun-2016	167	39	127	8-Jun-2016	1
127	1-Jun-2016	8-Jun-2016	167	39	128	15-Jun-2016	0
128	8-Jun-2016	15-Jun-2016	167	38	128	22-Jun-2016	1
129	15-Jun-2016	22-Jun-2016	167	39	128	29-Jun-2016	0
130	22-Jun-2016	29-Jun-2016	167	37	129	6-Jul-2016	1
131	29-Jun-2016	6-Jul-2016	167	37	129	13-Jul-2016	1
132	6-Jul-2016	13-Jul-2016	167	38	129	20-Jul-2016	0
133	13-Jul-2016	20-Jul-2016	167	38	129	27-Jul-2016	0
134	20-Jul-2016	27-Jul-2016	167	35	131	3-Aug-2016	1
135	27-Jul-2016	3-Aug-2016	167	35	132	10-Aug-2016	0
136	3-Aug-2016	10-Aug-2016	167	33	134	17-Aug-2016	0
137	10-Aug-2016	17-Aug-2016	167	32	135	24-Aug-2016	0
138	17-Aug-2016	24-Aug-2016	167	30	137	31-Aug-2016	0
139	24-Aug-2016	31-Aug-2016	167	28	139	7-Sep-2016	0
140	31-Aug-2016	7-Sep-2016	167	28	138	14-Sep-2016	1
141	7-Sep-2016	14-Sep-2016	167	29	138	21-Sep-2016	0
142	14-Sep-2016	21-Sep-2016	167	29	136	28-Sep-2016	2
143	21-Sep-2016	28-Sep-2016	167	30	137	5-Oct-2016	0
144	28-Sep-2016	5-Oct-2016	167	30	137	12-Oct-2016	0
145	5-Oct-2016	12-Oct-2016	167	30	136	19-Oct-2016	1
146	12-Oct-2016	19-Oct-2016	167	31	136	26-Oct-2016	0
147	19-Oct-2016	26-Oct-2016	167	29	137	2-Nov-2016	1
148	26-Oct-2016	2-Nov-2016	167	30	136	9-Nov-2016	1
149	2-Nov-2016	9-Nov-2016	167	31	136	16-Nov-2016	0
150	9-Nov-2016	16-Nov-2016	167	31	136	23-Nov-2016	0
151	16-Nov-2016	23-Nov-2016	167	31	135	30-Nov-2016	1
152	23-Nov-2016	30-Nov-2016	167	32	133	7-Dec-2016	2
153	30-Nov-2016	7-Dec-2016	167	33	132	14-Dec-2016	2
154	7-Dec-2016	14-Dec-2016	167	27	140	21-Dec-2016	0
155	14-Dec-2016	21-Dec-2016	167	27	139	28-Dec-2016	1
156	21-Dec-2016	28-Dec-2016	167	28	138	4-Jan-2017	1
157	28-Dec-2016	4-Jan-2017	167	29	138	11-Jan-2017	0
158	4-Jan-2017	11-Jan-2017	167	29	138	18-Jan-2017	0
159	11-Jan-2017	18-Jan-2017	167	29	137	25-Jan-2017	1
160	18-Jan-2017	25-Jan-2017	167	30	137	1-Feb-2017	0
161	25-Jan-2017	1-Feb-2017	167	30	136	8-Feb-2017	1
162	1-Feb-2017	8-Feb-2017	167	31	136	15-Feb-2017	0
163	8-Feb-2017	15-Feb-2017	167	31	135	22-Feb-2017	1
164	15-Feb-2017	22-Feb-2017	167	32	134	1-Mar-2017	1
165	22-Feb-2017	1-Mar-2017	167	32	134	8-Mar-2017	1
166	1-Mar-2017	8-Mar-2017	167	32	135	15-Mar-2017	0
167	8-Mar-2017	15-Mar-2017	167	31	136	22-Mar-2017	0
168	15-Mar-2017	22-Mar-2017	167	31	135	29-Mar-2017	1
169	22-Mar-2017	29-Mar-2017	167	32	134	5-Apr-2017	1
170	29-Mar-2017	5-Apr-2017	167	32	129	12-Apr-2017	6

171	5-Apr-2017	12-Apr-2017	167	37	129	19-Apr-2017	1
172	12-Apr-2017	19-Apr-2017	167	38	128	26-Apr-2017	1
173	19-Apr-2017	26-Apr-2017	167	39	127	3-May-2017	1
174	26-Apr-2017	3-May-2017	167	39	128	10-May-2017	0
175	3-May-2017	10-May-2017	167	38	129	17-May-2017	0
176	10-May-2017	17-May-2017	167	37	129	24-May-2017	1
177	17-May-2017	24-May-2017	167	36	131	31-May-2017	0
178	24-May-2017	31-May-2017	167	36	131	7-Jun-2017	0
179	31-May-2017	7-Jun-2017	167	35	132	14-Jun-2017	0
180	7-Jun-2017	14-Jun-2017	167	35	130	21-Jun-2017	2
181	14-Jun-2017	21-Jun-2017	167	36	131	28-Jun-2017	0
182	21-Jun-2017	28-Jun-2017	167	36	129	5-Jul-2017	2
183	28-Jun-2017	5-Jul-2017	167	38	128	12-Jul-2017	1
184	5-Jul-2017	12-Jul-2017	167	39	128	19-Jul-2017	0
185	12-Jul-2017	19-Jul-2017	167	39	126	26-Jul-2017	2
186	19-Jul-2017	26-Jul-2017	167	41	123	2-Aug-2017	3
187	26-Jul-2017	2-Aug-2017	167	42	125	9-Aug-2017	0
188	2-Aug-2017	9-Aug-2017	167	38	129	16-Aug-2017	0
189	9-Aug-2017	16-Aug-2017	167	37	130	23-Aug-2017	0
190	16-Aug-2017	23-Aug-2017	167	36	131	30-Aug-2017	0
191	23-Aug-2017	30-Aug-2017	167	35	132	6-Sep-2017	0
192	30-Aug-2017	6-Sep-2017	167	35	132	13-Sep-2017	0
193	6-Sep-2017	13-Sep-2017	167	35	131	20-Sep-2017	1
194	13-Sep-2017	20-Sep-2017	167	35	131	27-Sep-2017	1
195	20-Sep-2017	27-Sep-2017	167	36	130	4-Oct-2017	1
196	27-Sep-2017	4-Oct-2017	167	37	129	11-Oct-2017	1
197	4-Oct-2017	11-Oct-2017	167	37	129	18-Oct-2017	1
198	11-Oct-2017	18-Oct-2017	167	36	130	25-Oct-2017	1
199	18-Oct-2017	25-Oct-2017	167	37	129	1-Nov-2017	1
200	25-Oct-2017	1-Nov-2017	167	38	127	8-Nov-2017	2
201	1-Nov-2017	8-Nov-2017	167	40	126	15-Nov-2017	1
202	8-Nov-2017	15-Nov-2017	167	41	125	22-Nov-2017	1
203	15-Nov-2017	22-Nov-2017	167	42	123	29-Nov-2017	2
204	22-Nov-2017	29-Nov-2017	167	42	124	6-Dec-2017	1
205	29-Nov-2017	6-Dec-2017	167	41	125	13-Dec-2017	1
206	6-Dec-2017	13-Dec-2017	167	42	122	20-Dec-2017	3
207	13-Dec-2017	20-Dec-2017	167	45	121	27-Dec-2017	1
208	20-Dec-2017	27-Dec-2017	167	46	117	3-Jan-2018	4
209	27-Dec-2017	3-Jan-2018	167	50	117	10-Jan-2018	0
210	3-Jan-2018	10-Jan-2018	167	48	117	17-Jan-2018	2
211	10-Jan-2018	17-Jan-2018	167	50	115	24-Jan-2018	2
212	17-Jan-2018	24-Jan-2018	167	52	113	31-Jan-2018	2
213	24-Jan-2018	31-Jan-2018	167	54	112	7-Feb-2018	1
214	31-Jan-2018	7-Feb-2018	167	54	113	14-Feb-2018	0
215	7-Feb-2018	14-Feb-2018	167	54	113	21-Feb-2018	0
216	14-Feb-2018	21-Feb-2018	167	54	112	28-Feb-2018	1
217	21-Feb-2018	28-Feb-2018	167	55	112	7-Mar-2018	0
218	28-Feb-2018	7-Mar-2018	167	55	111	14-Mar-2018	1
219	7-Mar-2018	14-Mar-2018	167	55	112	21-Mar-2018	0
220	14-Mar-2018	21-Mar-2018	167	55	112	28-Mar-2018	0
221	21-Mar-2018	28-Mar-2018	167	54	111	4-Apr-2018	2
222	28-Mar-2018	4-Apr-2018	167	56	111	11-Apr-2018	0
223	4-Apr-2018	11-Apr-2018	167	56	109	18-Apr-2018	2
224	11-Apr-2018	18-Apr-2018	167	58	109	25-Apr-2018	0
225	18-Apr-2018	25-Apr-2018	167	58	109	2-May-2018	0
226	25-Apr-2018	2-May-2018	167	58	109	9-May-2018	0
227	2-May-2018	9-May-2018	167	58	109	16-May-2018	0
228	9-May-2018	16-May-2018	167	57	110	23-May-2018	0
229	16-May-2018	23-May-2018	167	54	113	30-May-2018	0
230	23-May-2018	30-May-2018	167	54	112	6-Jun-2018	1

231	30-May-2018	6-Jun-2018	167	53	113	13-Jun-2018	1
232	6-Jun-2018	13-Jun-2018	167	54	111	20-Jun-2018	2
233	13-Jun-2018	20-Jun-2018	167	56	110	27-Jun-2018	1
234	20-Jun-2018	27-Jun-2018	167	57	110	4-Jul-2018	0
235	27-Jun-2018	4-Jul-2018	167	57	110	11-Jul-2018	0
236	4-Jul-2018	11-Jul-2018	167	57	110	18-Jul-2018	0
237	11-Jul-2018	18-Jul-2018	167	56	111	25-Jul-2018	0
238	18-Jul-2018	25-Jul-2018	167	56	110	1-Aug-2018	1
239	25-Jul-2018	1-Aug-2018	167	52	115	8-Aug-2018	0
240	1-Aug-2018	8-Aug-2018	167	52	115	15-Aug-2018	0
241	8-Aug-2018	15-Aug-2018	167	51	116	22-Aug-2018	0
242	15-Aug-2018	22-Aug-2018	167	48	118	29-Aug-2018	1
243	22-Aug-2018	29-Aug-2018	167	45	122	5-Sep-2018	0
244	29-Aug-2018	5-Sep-2018	167	45	122	12-Sep-2018	0
245	5-Sep-2018	12-Sep-2018	167	45	122	19-Sep-2018	0
246	12-Sep-2018	19-Sep-2018	167	42	125	26-Sep-2018	0
247	19-Sep-2018	26-Sep-2018	167	41	126	3-Oct-2018	0
248	26-Sep-2018	3-Oct-2018	167	37	130	10-Oct-2018	0
249	3-Oct-2018	10-Oct-2018	167	36	131	17-Oct-2018	0
250	10-Oct-2018	17-Oct-2018	167	36	131	24-Oct-2018	0
251	17-Oct-2018	24-Oct-2018	167	36	129	31-Oct-2018	2
252	24-Oct-2018	31-Oct-2018	167	36	130	7-Nov-2018	1
253	31-Oct-2018	7-Nov-2018	167	36	131	14-Nov-2018	0
254	7-Nov-2018	14-Nov-2018	167	35	131	21-Nov-2018	1
255	14-Nov-2018	21-Nov-2018	167	36	129	28-Nov-2018	2
256	21-Nov-2018	28-Nov-2018	167	36	129	5-Dec-2018	2
257	28-Nov-2018	5-Dec-2018	167	38	129	12-Dec-2018	0
258	5-Dec-2018	12-Dec-2018	167	38	129	19-Dec-2018	0
259	12-Dec-2018	19-Dec-2018	167	38	129	26-Dec-2018	0

Number of participant farms could vary by week. The risk was estimated for the participant farms in the Morrison Swine Health Monitoring Project (MSHMP) of the University of Minnesota (https://vetmed.umn.edu/centers-programs/swine-program/outreach-leman-mshmp/mshmp, accessed on 20 March 2020).
